# Hypochromic red cells as a prognostic indicator of survival among patients with systemic sclerosis screened for pulmonary hypertension

**DOI:** 10.1186/s13075-023-03020-y

**Published:** 2023-03-09

**Authors:** Panagiota Xanthouli, Ojan Gordjani, Nicola Benjamin, Satenik Harutyunova, Benjamin Egenlauf, Alberto M. Marra, Simon Haas, Nicklas Milde, Norbert Blank, Hanns-Martin Lorenz, Christoph Fiehn, Silvia Ulrich, Oliver Distler, Ekkehard Grünig, Christina A. Eichstaedt

**Affiliations:** 1 Centre for Pulmonary Hypertension, Thoraxklinik Heidelberg gGmbH at Heidelberg University Hospital, Röntgenstrasse 1, D-69126 Heidelberg, Germany; 2 Translational Lung Research Centre Heidelberg (TLRC), Member of the German Centre for Lung Research (DZL), Heidelberg, Germany; 3 Department of Pneumology and Critical Care Medicine, Thoraxklinik Heidelberg gGmbH at Heidelberg University Hospital, Heidelberg, Germany; 4 Division of Rheumatology, Department of Internal Medicine V: Hematology, Oncology and Rheumatology, University Hospital Heidelberg, Heidelberg, Germany; 5 Department of Translational Medical Sciences, “Federico II” University and School of Medicine, Naples, Italy; 6 Unit for Rheumatology and Clinical Immunology, Medical Centre Baden-Baden, Baden-Baden, Germany; 7 Department of Pulmonology, University Hospital Zurich, University of Zurich, Zurich, Switzerland; 8 Department of Rheumatology, University Hospital Zurich, University of Zurich, Zurich, Switzerland; 9 Laboratory for Molecular Genetic Diagnostics, Institute of Human Genetics, Heidelberg University, Heidelberg, Germany

**Keywords:** Systemic sclerosis, Hypochromic erythrocytes, Hypochromic red cells, Iron deficiency, Prognosis

## Abstract

**Background:**

Patients with systemic sclerosis (SSc) are frequently affected by iron deficiency, particularly those with pulmonary hypertension (PH). The first data indicate the prognostic importance of hypochromic red cells (% HRC) > 2% among patients with PH. Hence, the objective of our study was to investigate the prognostic value of % HRC in SSc patients screened for PH.

**Methods:**

In this retrospective, single-center cohort study, SSc patients with a screening for PH were enrolled. Clinical characteristics and laboratory and pulmonary functional parameters associated with the prognosis of SSc were analyzed using uni- and multivariable analysis.

**Results:**

From 280 SSc patients screened, 171 could be included in the analysis having available data of iron metabolism (81% female, 60 ± 13 years of age, 77% limited cutaneous SSc, 65 manifest PH, and 73 pulmonary fibrosis). The patients were followed for 2.4 ± 1.8 (median 2.4) years. HRC > 2% at baseline was significantly associated with worse survival in the uni- (*p* = 0.018) and multivariable (*p* = 0.031) analysis independent from the presence of PH or pulmonary parenchymal manifestations. The combination of HRC > 2% and low diffusion capacity for carbon monoxide (DLCO) ≤ 65% predicted was significantly associated with survival (*p* < 0.0001).

**Conclusion:**

This is the first study reporting that HRC > 2% is an independent prognostic predictor of mortality and can possibly be used as a biomarker among SSc patients. The combination of HRC > 2% and DLCO ≤ 65% predicted could serve in the risk stratification of SSc patients. Larger studies are required to confirm these findings.

## Background

Iron deficiency (ID) is common among patients with autoimmune and chronic inflammatory disorders and was shown to be associated with worse prognosis particularly among patients with systemic sclerosis (SSc) [1]. Anemia occurs in up to 25% of SSc patients, mostly due to blood loss from their gastrointestinal tract, chronic disease, malabsorption syndrome, or hemolysis in case of renal crisis [2]. In clinical routine, ferritin and transferrin saturation are the most commonly used parameters for the diagnosis of ID [3]. The identification of ID among patients with chronic inflammatory disorders is complicated, as both parameters can be affected by inflammation, potentially resulting in misleading results [4] and a delay of ID diagnosis in a stage of advanced anemia [5]. Furthermore, serum iron levels fluctuate, depending on the nutrition and time point of the day [6]. Therefore, more robust biomarkers of the current iron status are needed to simplify the diagnostic process, leading to adequate and prompt treatment decisions.

The percentage of hypochromic erythrocytes or red cells (% HRC) was shown to be one of the most reliable markers for early identification of functional ID, as it reflects the iron status of the previous three months [6]. It was successfully used for the observation of functional ID in rheumatoid arthritis [7] and for monitoring of therapeutic response to iron supplementation in advanced chronic kidney disease [8]. Furthermore, previous data indicate that HRC > 2% could be relevant for the prognosis of patients with pulmonary arterial hypertension (PAH) [9].

In this study, we therefore aimed firstly to investigate the correlation of % HRC, as a marker of iron storage, with survival among patients with SSc in our cohort of patients screened for pulmonary hypertension (PH). Secondly, we sought to identify its association with further iron-related parameters as well as other pulmonary parenchymal manifestations of SSc.

## Methods

### Study population

In this single-center, retrospective cohort study, SSc patients were included, who were referred by their rheumatologists for a screening for PH at the expert center for PH at the Thoraxklinik Heidelberg gGmbH at Heidelberg University Hospital, Germany. SSc patients were sent with a suspicion of PH due to symptoms and laboratory or lung functional abnormalities from 2010 to 2020. The results from a part of this cohort were published before [10]. The criteria for SSc classification by the American College of Rheumatology/European League Against Rheumatism were fulfilled by all patients [11]. Patients were divided into limited (lcSSc) or diffuse cutaneous SSc (dcSSc) according to LeRoy’s criteria [12]. Patients were not included if they were underaged, not able to provide informed consent, had connective tissue diseases other than SSc, or had no data of % HRC at baseline. The routine assessment of % HRC in our clinical laboratory began in July 2014; therefore, patients assessed before could not be included in the analysis unless % HRC was determined externally.

There was no objection against the study from the ethics committee of the Medical Faculty of Heidelberg University Hospital (internal number S-126/2021). The study complied with the current version of the Declaration of Helsinki.

### Study design

Clinical and laboratory data from SSc patients’ files including the routinely performed assessments at first evaluation at the PH center as well as survival were analyzed. All patients received a standardized clinical workup including medical history, clinical examination, and detailed blood work. Laboratory examinations included liver and renal function parameters, inflammatory markers (i.e., C-reactive protein (CRP)), N-terminal pro-brain natriuretic peptide levels (NTproBNP), and analysis for ID including parameters from complete blood count such as hemoglobin, mean corpuscular hemoglobin (MCH), mean erythrocyte corpuscular volume (MCV), mean corpuscular hemoglobin concentration (MCHC), and % HRC, as well as ferritin and serum iron. HRC were characterized by erythrocytes containing < 28 g/dl hemoglobin. The normal reference range of % HRC in our laboratory was 0–2%. Furthermore, pulmonary function tests (PFT), electrocardiogram, World Health Organization functional class assessment (WHO-FC), 6-min walking distance (6MWD) test under standardized conditions [13], echocardiography at rest, high-resolution computed tomography scan of the lungs (HRCT), and right heart catheterization (RHC) at rest were performed. The presence of cardiac disease in the past history such as coronary heart disease assessed with left heart catheterization and/or pulmonary disease (i.e., PH and/or interstitial lung disease (ILD)) was documented. ILD was diagnosed in case of the presence of significant interstitial fibrosis on HRCT of the lungs (> 20% of parenchyma) or restrictive pattern in PFT in case of missing validation in HRCT (maximal vital capacity < 70% predicted with a concomitant normal ratio of forced expiratory volume in the first second to vital capacity). Manifest PAH was diagnosed according to the valid hemodynamic criteria, at the time, with a mean pulmonary arterial pressure (mPAP) ≥ 25 mmHg, pulmonary vascular resistance (PVR) > 3 Wood units (WU), and pulmonary arterial wedge pressure (PAWP) ≤ 15 mmHg measured by RHC [14]. The presence of pulmonary vascular disease (PVD) was defined as either mPAP 21–24 mmHg with PVR ≥ 2 WU or mPAP ≥ 25 mmHg [10].

### Statistical analyses

Descriptive statistics were used to present the patients’ characteristics and clinical parameters with mean ± standard deviation or frequency. The chi-square test was employed for frequency data, presented as *n* and %.

The threshold for anemia was hemoglobin < 12 g/dl in female patients and < 13 g/dl in male patients. ID was defined by the threshold for ferritin as < 30 ng/ml with normal levels of CRP and ferritin < 100 ng/ml with CRP levels > 5 mg/l. The reference cutoffs were based on reference ranges that were either previously established by clinical research or taken from the local laboratory.

The relevance for survival was investigated for clinical parameters associated with outcome in SSc including sex, age, type of SSc, presence of ILD [15], DLCO ≤ 65% predicted [5], and PVR ≥ 2 WU [10]. Furthermore, parameters associated with iron metabolism or inflammation including CRP and white blood cell count were compared between the groups. The Mann-Whitney *U* test was employed to compare the clinical characteristics between the patient groups with HRC > 2% and ≤ 2%.

The prognostic values for survival were investigated by uni- and multivariable Cox regression analysis. Death due to any cause, date of lung transplantation, or date of last contact was recorded for survival at follow-up and compared to the time of the first evaluation (baseline). Univariable categorial analysis was performed by Kaplan-Meier analysis. A multivariable Cox model was performed including all variables, which were significantly associated with survival (*p* < 0.05) in the univariable log rank tests. The combined independent parameters for risk assessment were compared with known risk stratification tools REVEAL [16], REVEAL 2.0 [17], COMPERA [18], and the French risk assessment strategy [19]. *p*-values < 0.05 were considered as statistically significant. IBM SPSS V 27.0 was used to conduct all analyses (IBM Corp. Released 2020. IBM SPSS Statistics for Macintosh, version 27.0. Armonk, NY: IBM Corp.).

## Results

### Patients’ characteristics

**Fig. 1:**
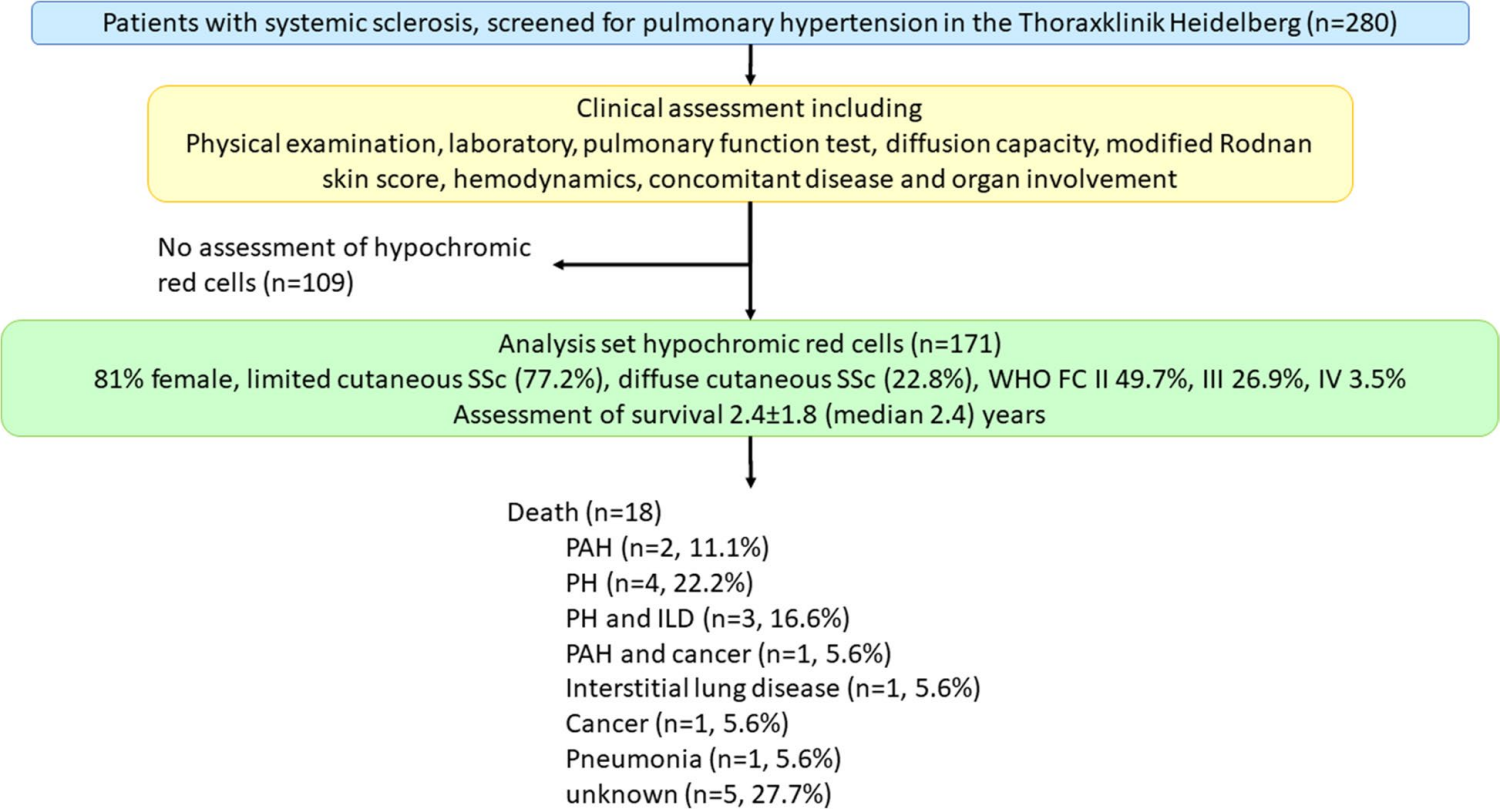
Study flowchart. Out of 280 patients, 171 had an assessment of hypochromic erythrocytes at the initial screening for pulmonary hypertension. Patients were assessed clinically and followed for 2.4 ± 1.8 (median 2.4) years

Of 280 SSc patients screened for PH at the Centre for Pulmonary Hypertension, Thoraxklinik Heidelberg, 171 patients could be included (Fig. 1). The baseline characteristics of the patients of the whole study cohort are detailed in Table 1. Out of the 171 patients, 139 were female (81.3%), the mean age was 60 ± 13 years, 132 patients (77%) had lcSSc, and 39 had dcSSc (23%). The mean SSc duration was 8.3 ± 9.4 years. Pulmonary arterial pressure elevation was present in 106 patients (62%), 41 were diagnosed with associated PAH (24%), and 65 had pre-capillary PH (38%) with mPAP ≥ 25 mmHg and PAWP ≤ 15 mmHg. ILD was detectable in 73 patients (43%). All patients with SSc-associated PAH received targeted PAH therapy according to the previous PH guidelines [14]. Of the 171 patients included, 50% were in WHO-FC II, 27% in WHO-FC III, and 4% in WHO-FC IV. The mean 6MWD was 428 ± 103 m. Overall, 40 patients (23%) suffered from coronary artery disease and 67 (39%) from systemic arterial hypertension. The renal function was normal on average. The mean C-reactive protein was 6.0 ± 9.1 mg/l, with 52 (30.4%) patients having a CRP > 5 mg/l.

**Table 1:** Characteristics of the patients at baseline

Parameter [unit]	Whole cohort (***n*** = 171)				
	Mean or ***n***	±	SD or (%)	95%CI	***n***
Age [years]	60.15	±	13.16	58.16 to 62.13	
Height [cm]	165.56	±	8.31	164.30 to 166.81	
Weight [kg]	69.84	±	15.62	67.48 to 72.20	
Female sex, no. [%]	139		81.3%		
**World Health Organization functional class**					
I	34		19.9%		
II	85		49.7%		
III	46		26.9%		
IV	6		3.5%		
**SSc subgroups**					
Limited cutaneous SSc	132		77.2%		
Diffuse cutaneous SSc	39		22.8%		
**SSc disease duration [years]**	8.31	±	9.38	6.88 to 9.73	168
**Modified Rodnan Skin Score**	10.99	±	11.01	8.74 to 13.24	94
**Oxygen saturation [%]**	95.66	±	3.44	95.14 to 96.18	
**Digital ulcers**	65		39.4%		165
**Arterial hypertension**	67		39.4%		170
**Pulmonary fibrosis**	73		42.7%		
**Hemodynamics at rest**					
mPAP [mmHg]	23.84	±	10.12	22.31 to 25.37	
PAWP [mmHg]	10.45	±	4.21	9.82 to 11.09	170
Cardiac output [l/min]	5.29	±	1.58	5.05 to 5.53	167
Cardiac index [l/min/m^2^]	3.06	±	0.85	2.93 to 3.19	169
PVR [WU]	2.80	±	2.19	2.47 to 3.13	170
**Echocardiography at rest**					
Right atrial area [cm^2^]	13.45	±	4.80	12.71 to 14.19	164
Right ventricular area [cm^2^]	15.29	±	4.21	14.64 to 15.94	164
sPAP [mmHg]	35.20	±	14.48	32.97 to 37.43	164
TAPSE [mm]	23.61	±	4.88	22.86 to 24.36	165
**Lung function**					
VCmax [%]	92.03	±	23.74	88.39 to 95.67	166
FEV1 [l]	2.26	±	0.71	2.15 to 2.37	166
TLC [l]	5.03	±	1.21	4.84 to 5.21	166
DLCO [%]	56.88	±	19.71	53.73 to 60.03	153
DLCO/VA [%]	68.25	±	20.83	64.96 to 71.54	156
**Laboratory**					
NTproBNP [ng/l]	584.37	±	1487.97	353.51 to 815.24	162
WBC [/nl]	7.73	±	2.62	7.33 to 8.12	
Creatinine [mg/dl]	0.84	±	0.28	0.80 to 0.88	
GFR [ml/min/1.73 m^2^]	86.20	±	27.56	82.03 to 90.37	170
MCH [pg]	29.89	±	2.02	29.59 to 30.20	
MCV [fl]	90.00	±	5.31	89.20 to 90.80	
CRP [mg/l]	6.04	±	9.08	4.67 to 7.41	
Ferritin [ng/ml]	103.66	±	128.38	83.68 to 123.46	161
Hemoglobin [g/dl]	13.37	±	1.40	13.16 to 13.58	
Iron [μmol/l]	12.95	±	6.01	12.01 to 13.88	161
**Presence of autoantibodies**					146
Anti-Scl-70	48		32.9%		
CENT	52		35.6%		
Others	46		31.5%		
**6-min walking distance [m]**	428.23	±	103.15	411.65 to 444.82	151

*mPAP* mean pulmonary arterial pressure, *PAWP* pulmonary arterial wedge pressure, *PVR* pulmonary vascular resistance, *WU* Wood units, *sPAP* systolic pulmonary arterial pressure, *TAPSE* tricuspid annular plane systolic excursion, *VC* vital capacity, *FEV1* forced expiratory volume in first second, *TLC* total lung capacity, *DLCO* diffusion capacity of carbon monoxide, *DLCO/VA* diffusion capacity of carbon monoxide divided by the alveolar volume, *NTproBNP* N-terminal pro-brain natriuretic peptide, *WBC* white blood cells, *GFR* glomerular filtration rate, *MCH* mean corpuscular hemoglobin, *MCV* mean corpuscular volume, *CRP* C-reactive protein, *Anti-Scl-70* anti-topoisomerase I antibodies, *CENT* anti-centromere antibodies, *Others* mostly RNA-polymerase I/II/II, antinuclear antibodies (ANA), fibrillarin, Pm/Scl

### Iron status and anemia

At baseline, anemia was present in 17 patients (10%), and 13 of them were females. ID was identified among 59 patients (35%). Thirty-eight patients (22%) received iron supplementation during follow-up. As ID could be caused by malignancies and gastrointestinal bleeding especially among SSc patients, we examined the patients’ medical records for any such events. Overall, malignancies were reported in 16 patients (9%). Most of them (11/16) had had a tumor long before the diagnosis of SSc and had no signs of relapse during the first presentation and evaluation of iron status in our clinic, whereas five patients developed a malignancy 4 to 5 years after the first screening. The tumors reported included breast cancer (7 patients), gastrointestinal tumors (3 patients), and others (6 patients). Gastrointestinal bleeding was identified in 6 patients, at least 1 year after the first evaluation, and none suffered from anemia at baseline.

### Survival of SSc patients

The patients were followed for 2.4 ± 1.8 (median 2.4) years. During the observation time, 18 patients died (Fig. 1), 12 due to pulmonary complications (mostly P(A) H and/or ILD, one due to breast cancer and PAH, and one due to pneumonia and sepsis); one patient died due to lung cancer; and for 5 patients (27.7%), the cause remained unknown (Fig. 1). The mean estimated survival was 7.3 ± 0.5 (standard error of the mean) years from baseline (date of screening assessment).

#### Predictors of survival

**Fig. 2:**
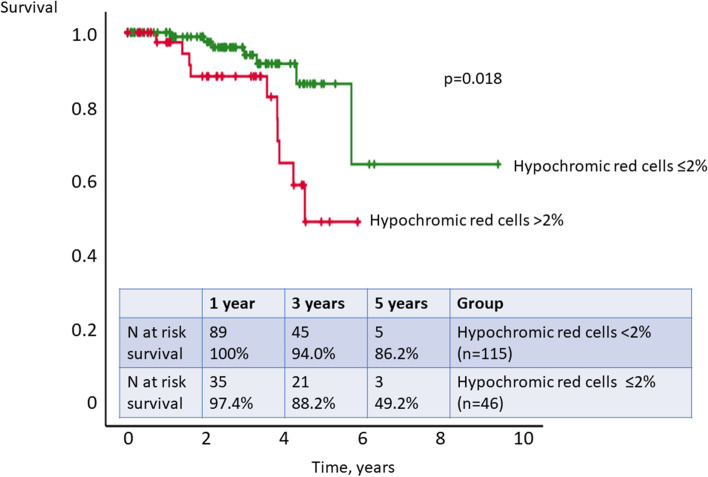
Kaplan-Meier analysis of hypochromic red cells > 2% vs. ≤ 2%. Hypochromic erythrocytes > 2% significantly predicted survival in the study cohort (*p* = 0.018)

**Fig. 3:**
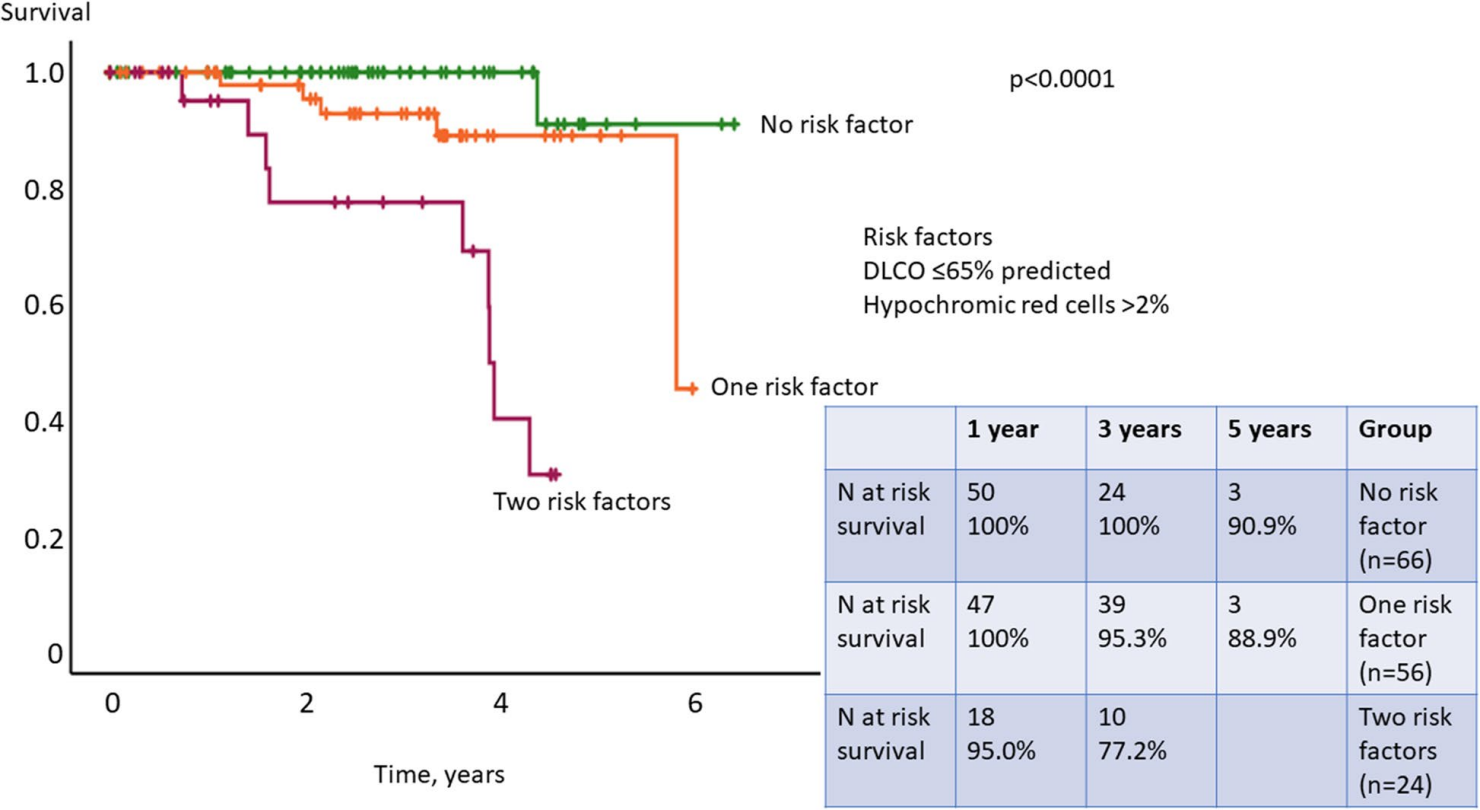
Kaplan-Meier analysis of the multivariable risk set. DLCO ≤ 65% predicted and hypochromic red cells > 2% were identified as independent prognostic predictors (*p* < 0.0001)

In order to investigate the parameters associated with survival, parameters correlating with iron status as well as further known prognostic predictors for survival in SSc were assessed using Cox regression analysis. In the univariable analysis analyzing parameters of iron metabolism, only % HRC was significantly associated with survival (*p* = 0.043). Neither ferritin nor hemoglobin showed prognostic value for survival (*p* = 0.353 and *p* = 0.290, respectively). In the categorial univariable analysis performed with Kaplan-Meier analysis, HRC > 2% was significantly associated with worse survival (*p* = 0.018, Fig. 2). In the univariable analysis investigating the known prognostic parameters in SSc, DLCO ≤ 65% predicted, age ≥ 60 years at baseline, and PVR ≥ 2 WU were significantly associated with survival in our cohort (*p* < 0.001, *p* = 0.040, and *p* = 0.041, respectively). The autoantibody status showed no correlation with survival (*p* = 0.131). In the multivariable Cox regression analysis, only two factors, HRC > 2% (*p* = 0.031) and low diffusion capacity for carbon monoxide (DLCO) ≤ 65% predicted (*p* = 0.013), were independent prognostic factors for survival (Table 2). Since ILD and PVR ≥ 2 WU did not significantly improve the multivariable model, the two parameters HRC > 2% and DLCO ≤ 65% predicted were independent from the presence of pulmonary fibrosis or pulmonary vascular pressure increase in this cohort of SSc patients. The combination of HRC > 2% and DLCO ≤ 65% predicted was significantly associated with mortality (*p* < 0.0001, Table 2). The two independent prognostic predictors of the multivariable analysis (identified by stepwise forward selection with likelihood ratio), HRC > 2% and DLCO ≤ 65% predicted, were analyzed by Kaplan-Meier analysis (*p* < 0.0001). In the presence of both risk factors, the 1- and 3-year survival was 95.0% and 77.2%, respectively; in the presence of only one risk factor, the 1- and 3-year survival was 100% and 95.3%, respectively; and in the absence of both risk factors, the 3-year survival was 100% (Kaplan-Meier, *p* < 0.0001, Fig. 3).

**Table 2:** Predictors of survival in the uni- and multivariable analysis

Variables	Univariable analysis		Multivariable Cox regression analysis	
	***p***-value	***n***	***p***-value	***n***
**Hypochromic erythrocytes**	0.043	170		
**White blood cell count**	0.298	170		
**Ferritin**	0.353	160		
**Iron**	0.702	160		
**Hemoglobin**	0.290	170		
**MCH**	0.301	170		
**MCV**	0.143	170		
**Categorial**^a^				
MCV ≤ 80 fl	0.920	163		
Hypochromic erythrocytes > 2%	0.018	163	0.031	145
**Known prognostic predictors**				
Sex	0.322	163		
Type of SSc	0.679	163		
DLCO ≤ 65% predicted	< 0.001	149	0.013	145
ILD presence	0.066	163		
Age ≥ 60 years, baseline	0.040	160		
PVR ≥ 2 WU	0.041	161		

*MCV* mean corpuscular volume, *SSc* systemic sclerosis, *DLCO* diffusion capacity of the lung for carbon monoxide, *ILD* interstitial lung disease, *PVR* pulmonary vascular resistance

^a^Univariable categorical analyses were performed with Kaplan-Meier analysis, and all other analyses were Cox regression analyses

In comparison with other risk stratification tools (REVEAL, REVEAL 2.0, COMPERA, and French approach), the combination of HRC > 2% and DLCO ≤65% predicted was superior to discriminate survival (Cox regression *p* < 0.0001).

### Distinction of patients with HRC ≤ 2% and > 2%

Patients were divided into two groups according to % HRC (Table 3). Patients with HRC > 2% showed a more severely impaired PFT with significantly reduced lung volumes and lower diffusion capacity (Table 3). There was no difference in terms of renal function between the groups. Patients with HRC > 2% had significantly lower hemoglobin and iron levels as well as lower MCH (*p* < 0.001, *p* = 0.011, *p* = 0.001, respectively), although ferritin levels did not significantly differ between the groups (*p* = 0.857). The inflammatory marker CRP was higher in patients with HRC > 2% (*p* = 0.031), but the leukocyte count showed no difference (*p* = 0.622) between the groups. No correlations could be identified for HRC > 2% regarding age, gender, presence of ILD, or SSc subtype (*p* = 0.432, *p* = 0.193, *p* = 0.121, and *p* = 0.903, respectively).

**Table 3:** Baseline characteristics of patients with HRC ≤ 2% or > 2%

Parameter [unit]	Cohort with HRC% ≤ 2% (***n*** = 123)				Cohort with HRC% > 2% (***n*** = 48)				***p***
	Mean or ***n***	±	SD or (%)	***n***^a^	Mean or ***n***	±	SD or (%)	***n***^a^	
**Age [years]**	59.65	±	12.93		61.42	±	13.81		0.432
**Height [cm]**	166.7	±	8.38		162.63	±	7.44		0.004
**Weight [kg]**	71.18	±	16.06		66.4	±	14.01		0.072
**Female sex, no. [%]**	97		78.9%		42		87.5%		0.193
**World Health Organization functional class**									0.017
I	25		20.3%		9		18.8%		
II	69		56.1%		16		33.3%		
III	26		21.2%		20		41.7%		
IV	3		2.4%		3		6.3%		
**SSc subgroups**									0.903
Limited cutaneous SSc	96		78.1%		36		75.0%		
Diffus cutaneous SSc	27		22.0%		12		25.0%		
**SSc disease duration [years]**	8.18	±	9.43	121	8.66	±	9.35	47	0.765
**Modified Rodnan Skin Score**	10	±	11	67	14	±	11	27	0.114
**Oxygen saturation [%]**	95.96	±	3.29		94.9	±	3.75	48	0.071
**Digital ulcers**	43		35.8%	120	22		48.9%	45	0.136
**Arterial hypertension**	46		37.7%	122	21		43.8%		0.468
**Interstitial lung disease**	48		39.0%		25		52.1%		0.121
**Hemodynamics at rest**									
mPAP [mmHg]	22.25	±	9.16		27.92	±	11.35		0.003
PAWP [mm Hg]	10.42	±	4.46		10.55	±	3.50	47	0.848
Cardiac output [l/min]	5.40	±	1.74	121	4.99	±	1.05	46	0.065
Cardiac index [l/min/m^2^]	3.01	±	0.82	122	3.18	±	0.93	47	0.265
PVR [WU]	2.39	±	1.74	122	3.84	±	2.82		0.002
**Echocardiography at rest**									
Right atrial area [cm^2^]	13.03	±	4.25	117	14.48	±	5.88	47	0.131
Right ventricular area [cm^2^]	15.05	±	4.08	117	15.89	±	4.52	47	0.246
sPAP [mmHg]	32.45	±	11.84	116	41.85	±	17.90		0.001
TAPSE [mm]	23.98	±	4.53	117	22.70	±	5.59		0.161
**Lung function**									
VCmax [%]	95.05	±	22.72	121	83.92	±	31.02	45	0.007
FEV1 [l]	2.39	±	0.72	121	1.90	±	0.56	45	<0.001
TLC [l]	5.21	±	1.21	121	4.54	±	1.08	45	0.001
DLCO [%]	59.36	±	19.61	114	49.65	±	18.36	39	0.008
DLCO/VA [%]	70.97	±	19.46	115	60.63	±	22.82	41	0.006
**Laboratory**									
NTproBNP [ng/l]	465.53	±	1452.81	116	884.07	±	1548.96	46	0.107
WBC [/nl]	7.79	±	2.41		7.57	±	3.12		0.622
Creatinine [mg/dl]	0.836	±	0.281		0.846	±	0.271		0.826
GFR [ml/min/1.73 m^2^]	86.61	±	24.90	122	85.17	±	33.67		0.789
Urea [mg/dl]	34.3	±	16.7		34.79	±	21.00		0.873
MCH [pg]	30.27	±	1.61		28.93	±	2.59		0.001
MCV [fL]	90.48	±	4.58		88.77	±	6.73		0.11
Troponin T [pg/ml]	11.21	±	13.01	101	11.83	±	10.24	38	0.792
CRP [mg/l]	4.69	±	5.04	123	9.50	±	14.68		0.031
Ferritin [ng/ml]	104.82	±	111.74	115	100.77	±	164.20	46	0.857
Hemoglobin [g/dl]	13.73	±	1.24		12.46	±	1.38		< 0.001
Iron [μmol/l]	13.69	±	5.05	116	11.03	±	7.71	45	0.011
**Presence of autoantibodies**				104				42	0.333
Anti-Scl-70	38		36.6%		10		23.8%		
CENT	35		33.6%		17		40.5%		
Others	31		29.8%		15		35.7%		
**6-min walking distance [m]**	446.44	±	100.35	111	377.7	±	94.63	40	< 0.001

*mPAP* mean pulmonary arterial pressure, *PAWP* pulmonary arterial wedge pressure, *PVR* pulmonary vascular resistance, *WU* Wood units, *sPAP* systolic pulmonary arterial pressure, *TAPSE* tricuspid annular plane systolic excursion, *VC* vital capacity, *FEV1* forced expiratory volume in first second, *TLC* total lung capacity, *DLCO* diffusion capacity of carbon monoxide, *DLCO/VA* diffusion capacity of carbon monoxide divided by the alveolar volume, *NTproBNP* N-terminal pro-brain natriuretic peptide, *WBC* white blood cells, *GFR* glomerular filtration rate, *MCH* mean corpuscular hemoglobin, *MCV* mean corpuscular volume, *CRP* C-reactive protein, *Anti-Scl-70* anti-topo-isomerase I antibodies, *CENT* anti-centromere antibodies, *Others* mostly RNA-polymerase I/II/II, antinuclear antibodies (ANA), fibrillarin, Pm/Scl

^a^*n* is provided in case of missing values

### Hypochromic red cells in pulmonary vascular disease (PVD)

Patients with > 2% HRC displayed a significantly more severe hemodynamic impairment with significantly higher mPAP and PVR values (*p* = 0.003 and 0.002, respectively). In transthoracic echocardiography at rest, they presented with significantly higher systolic pulmonary arterial pressures (*p* = 0.001), although there were no differences in right heart size or function between the groups. Furthermore, patients with HRC > 2% were more severely physically impaired and had significantly shorter 6MWD (*p* < 0.001) and worse WHO-FC (chi-square *p* = 0.017). The occurrence of PVD during follow-up could be predicted with a sensitivity of 65% and a specificity of 83.5% in the presence of the independent risk factors HRC > 2% and/or DLCO ≤ 65% predicted.

## Discussion

This is the first study investigating the importance of % HRC as a marker of deranged iron supply demonstrating that a concentration of more than 2% of HRC is a strong predictor for worse survival in SSc patients independent from the presence of ILD and/or PH. On the contrary, ferritin, hemoglobin, and serum iron as conventional parameters of ID were not associated with mortality in our cohort. Moreover, the combination of HRC > 2% and DLCO ≤ 65% predicted was shown to be a useful tool to stratify patients at higher mortality risk (*p* < 0.0001). Additionally, the combination of these factors could be useful to predict the development of PVD during follow-up with a sensitivity of 65% and a specificity of 83.5% in our cohort.

### Iron deficiency and HRC as a prognostic indicator in SSc

Iron deficiency can be present in up to 25% of SSc patients even without anemia [20] and was associated with worse survival especially among SSc patients with PVD [1]. Mainly ferritin, MCV, and soluble transferrin receptor (sTfR) are traditional markers for ID [21]. However, the predictive value of these parameters for ID is limited especially in inflammatory diseases. Ferritin is an acute-phase protein and thus fluctuates due to inflammation [4]. This could explain why ferritin was not associated with outcomes among patients in our cohort, and there was no difference in ferritin levels among the groups with high or low % HRC. Furthermore, hemoglobin and other iron-related parameters such as serum iron, MCV, and MCH were not associated with survival in the uni- and multivariable analysis in contrast to % HRC. Being less influenced by inflammation [22], % HRC may provide a valuable biomarker of iron status in SSc. It not only correlated with significantly worse survival independent from the presence of pulmonary manifestations, but also with the development of PVD when combined with low DLCO.

#### Risk stratification model: HRC and DLCO

Previous studies showed that reduced DLCO, as a parameter to estimate the pulmonary capability to transfer oxygen to erythrocytes, was associated with worse survival in SSc patients [5]. We showed that the percentage of red blood cells containing < 28 g/dl hemoglobin or having a MCH < 28 pg was associated with worse survival in SSc patients. In the multivariable analysis, only DLCO ≤ 65% predicted and HRC > 2% were independent prognostic predictors for survival. The combination of these parameters could also predict the development of PVD with a sensitivity of 65% and a specificity of 83.3%. The latter indicates that patients with both risk factors should be closely monitored for the development of PVD at follow-up constituting a simple risk assessment model.

### Strengths and limitations

Our study included a large cohort of SSc patients being screened for PH. It provided valuable insights into the prognosis and stratification in this patient population with a rare disease. The patients underwent a detailed clinical work-up in an expert center including thorough diagnostics for the presence of any pulmonary SSc-associated involvement, including lung imaging by HRCT and invasive assessment for potential PVD by RHC. The presence of a higher proportion of HRC and its association with worse prognosis stresses the need for a thorough evaluation of these patients to identify those with ID. Furthermore, the calculation of % HRC is included in the clinical routine measurements in normal blood work and is cheap, well-established, easily, and promptly estimated.

On the other hand, due to the retrospective character of our study, the interpretation of further parameters of iron metabolism was limited as measurements such as transferrin saturation and soluble transferrin receptor were often missing from the routine work-up. The presence of gastrointestinal involvement was furthermore based on information from the past medical history according to the patients’ records and not based on endoscopic exclusion. Moreover, the inclusion of patients receiving a screening for PH in a specialized center for pulmonary diseases might have led to biased data. Due to the single-center nature of this study, the applicability of results to other cohorts is unclear, and thorough future investigations including further centers is warranted.

Prospective studies examining ID parameters including % HRC and the effect of parenteral iron supplementation on improvement of quality of life, exercise capacity, and survival might be useful in the future.

## Conclusions

The presence of HRC > 2% was independently associated with impaired survival among patients with SSc. The presence of low DLCO and high HRC added predictive power not only for survival but also for the possible development of impairment in pulmonary vasculature leading to early signs of PVD. Thus, this new biomarker might serve as a parameter for the risk assessment of SSc patients. Furthermore, % HRC might be used to measure functional ID more accurately and independently from the presence of inflammation than the commonly employed parameters ferritin, transferrin, and transferrin saturation. HRC may serve as a biomarker for the indication of iron substitution and for monitoring of iron status. Larger studies to validate the importance of this biomarker in clinical daily routine and therapeutic application are warranted.

## Data Availability

The datasets are available upon reasonable request to the corresponding author.

## Abbreviations

CRP: C-reactive protein
dcSSc: Diffuse cutaneous systemic sclerosis
DLCO: Diffusion capacity of carbon monoxide
FC: Functional class
HRC: Hypochromic red cells (erythrocytes)
HRCT: High-resolution computed tomography
ID: Iron deficiency
lcSSc: Limited cutaneous systemic sclerosis
MCH: Mean corpuscular hemoglobin
MCHC: Mean corpuscular hemoglobin concentration
MCV: Mean corpuscular volume
mPAP: Mean pulmonary arterial pressure
NTproBNP: N-terminal pro-brain natriuretic peptide levels
PAH: Pulmonary arterial hypertension
PAWP: Pulmonary arterial wedge pressure
PFT: Pulmonary function test
PH: Pulmonary hypertension
PVD: Pulmonary vascular disease
PVR: Pulmonary vascular resistance
RHC: Right heart catheterization
SSc: Systemic sclerosis
sTfR: Soluble transferrin receptor
WHO: World Health Organization
WU: Wood units
6MWD: 6-min walking distance
